# Assessment of Knowledge, Attitude, and Practice of HIV Positive Mothers on Antiretroviral Treatment towards Infant Feeding in Gondar Town Health Institutions, North West Ethiopia, 2017

**DOI:** 10.1155/2019/9107989

**Published:** 2019-01-01

**Authors:** Daniale Tekelia Ekubagewargies, Habtamu Sewunet Mekonnen, Tsehayu Melak Siyoum

**Affiliations:** ^1^Department of Pediatrics and Child Health Nursing, School of Nursing, College of Medicine and Health Sciences, University of Gondar, Ethiopia; ^2^Department of Medical Nursing, School of Nursing, College of Medicine and Health Sciences, University of Gondar, Ethiopia; ^3^Department of pediatrics, University of Gondar Specialized Hospital, Ethiopia

## Abstract

**Introduction:**

The world health organization recommends feeding practices for infants born from Human Immunodeficiency Virus infected mothers to be safe to both the infant and the mother. This includes prevention of mother to child transmission of the virus and at the same time meeting nutritional requirements of the child. This requires prioritizing prevention of HIV transmission through breastfeeding against non-HIV morbidity and mortality especially from malnutrition and serious illnesses such as diarrhea, among nonbreastfed infants.

**Objective:**

This study was aimed at assessing knowledge, attitude, and practice of HIV positive mothers on antiretroviral therapy towards infant feeding.

**Method:**

Institution based cross-sectional study was conducted among 402 HIV positive mothers at ART clinics of Gondar town from March 1 to April 18, 2017. Systematic random sampling technique was used to select study participants. Data was collected using a structured, pretested, interviewer-administered questionnaire. The collected data was entered into Epi Info version 7 and analyzed using SPSS version 20 software.

**Result:**

A total of 402 participants were interviewed with a 100% response rate. The mean age of participants was 29.24 (SD±10.06) years. The overall level of participant good knowledge and favorable attitude was 68.91% and 75.87%, respectively. Only 23.7% of mothers were practicing infant feeding according to WHO recommendation.

## 1. Background

The world health organization (WHO) recommends feeding practices for infants born from Human Immunodeficiency Virus (HIV) infected mothers to be safe to both the infant and the mother. This includes prevention of mother to child transmission (PMTCT) of the virus and at the same time meeting nutritional requirements of the child. This requires prioritizing prevention of HIV transmission through breastfeeding against non-HIV morbidity and mortality especially from malnutrition and serious illnesses such as diarrhea, among nonbreastfed infants [1].

In light of previous findings, it is recommended that HIV positive mothers use either exclusive breastfeeding (EBF) for 6 months of age or exclusive replacement feeding (ERF) if the latter is Acceptable, Feasible, Affordable, Sustainable, and Safe (AFASS) [2]. At six months of age, continuation of breastfeeding with additional complementary foods is recommended. Choice over breastfeeding or breast milk substitute depends on extended family or community, the nature of work and lifestyle of the mother or family [1]. All breastfeeding should stop once a nutritionally adequate and safe diet without breast milk can be provided [3].

It is estimated that the risk of mother to child transmission of HIV virus is 10-20% for those breastfeeding for two years [4, 5]. But, if exclusive breastfeeding is practiced, it is associated with a reduced risk of HIV transmission in the early months of postpartum, compared to mixed breastfeeding [6, 7].

HIV transmission through breastfeeding is pervasive in Sub-Saharan Africa, where mixed feeding is a predominant form of infant feeding as a result of a combination of cultural norms as well as economic conditions [5]. In this region, the majority of HIV positive mothers choose EBF but due to traditional factors and lack of practical support, the majority end up with mixed feeding resulting in high rates of postnatal HIV transmission [6].

In Ethiopia, 58% of children under the age of 6 months are exclusively breastfed regardless of maternal HIV status [8]. Despite few local studies conducted in different parts of the country, there is no literature that has tried to identify infant feeding practice among HIV positive mothers in the study area. Thus, the purpose of this study was to assess the existing infant feeding knowledge, attitude, and practices (KAP) among HIV positive mothers attending antiretroviral therapy (ART) clinics of Gondar town.

## 2. Methods

### 2.1. Study Area and Design

Institution based cross-sectional study was conducted from March 1 to April 18, 2017.

### 2.2. Study Area

This study was conducted in Gondar town government health institutions. Gondar town has eight health centers (clinics rendering service to about 5000 people of the catchment area) and one specialized hospital. These health institutions serve about four million people and have a combined average of 400 visiting patients per day. The number of people on ART in these health institutions is 12,562 and number of pregnant and lactating mothers on ART is 826.

### 2.3. Sample Size Determination

The sample size for the study was determined using the formula for single population proportion by assuming 5% marginal error and 95% confidence interval (*∂*(alpha)=0.5) and the prevalence (P) for KAP and infant feeding practices 38.8% [9].

(1) $$\begin{array}{c} \frac{{\left(1.96\right)}^{2}x0.388\left(1\text{-}0.0.388\right)}{{\left(0.5\right)}^{2}}=365 \end{array}$$

By assuming a 10% nonresponse rate, the final sample was 402.

### 2.4. Sampling Techniques

All health centers and Gondar university specialized hospital were studied. The sample size was allocated to health institutions proportionally based on the number of mothers on ART. Systematic random sampling technique was used for selecting individual participant mothers.

### 2.5. Inclusion Criteria

All HIV positive mothers on ART with babies aged 6–12 months were included in the study.

### 2.6. Exclusion Criteria

- Mothers who had difficulty of communication.
- Mothers who were severely ill.

### 2.7. Data Collection and Quality Control

Data quality was maintained by the following data quality control mechanisms; the questionnaire was pretested on 5% of the sample (i.e., 21 mothers on ART) at Felege-Hiwot referral hospital. One-day training was given to data collectors and supervisors. The questionnaire designed in English was translated to Amharic, the local language, and back to English for consistency. As this study used the interviewer-administered questionnaire, to reduce social desirability bias, data collectors who were not working in the respective health institutions were recruited. Strict supervision of the data collection was carried out throughout the data collection period. The collected data was checked for its consistency and completeness before any attempt to enter the code and analyze it.

### 2.8. Data Processing and Analysis

Raw data was cleaned, coded, entered to EPI Info version 7, and exported to SPSS version 20 for analysis. Frequency tables, means, and standard deviations were used to summarize the data. And finally, the result was interpreted to valuable information and presented using text and tabular form.

### 2.9. Operational Definitions

- Poor knowledge: If the respondent scores below the mean for knowledge questions.
- Good knowledge: If the respondent scores the mean or above for knowledge questions.
- Favorable Attitude: If the respondent scores the mean or above for attitude questions.
- Unfavorable attitude: If the respondent scores below the mean for attitude questions.
- Good practice: If the respondent complies with WHO recommendation of feeding of infants born to HIV positive mothers [1]. Either
  1. Exclusive breastfeeding, fulfilling: (a) initiating breastfeeding within the first hour of life; (b) exclusive breastfeeding for the first six months of life (infant only receives breast milk without any additional food or drink, not even water); followed by (c) continued breastfeeding for up to two years or beyond (with introduction of appropriate complementary foods at six months); and (d) breastfeeding on demand, that is, as often as the child wants, day and night or
  2. ERF (no breastfeeding at all): Mothers known to be living with HIV should only give commercial infant formula milk as a replacement feed to their HIV-uninfected infants or infants who are of unknown HIV status when specific conditions are met: (A) safe water and sanitation are assured at the household level and in the community; and (B) the mother or other caregiver can reliably provide sufficient infant formula milk to support the normal growth and development of the infant; and (C) the mother or caregiver can prepare it cleanly and frequently enough so that it is safe and carries a low risk of diarrhea and malnutrition; and (D) the mother or caregiver can, in the first six months, exclusively give infant formula milk; and (E) the family supports this practice; and (F) the mother or caregiver can access healthcare that offers comprehensive child health services. These descriptions are intended to give simpler and more explicit meaning to the concepts represented by AFASS (Acceptable, Feasible, Affordable, Sustainable, and Safe).
- Poor practice: If the respondent practices neither EBF nor ERF according to WHO recommendation.

## 3. Result

### 3.1. Sociodemographic Characteristics

A total of 402 participants were interviewed with a 100% response rate. The mean age of participants was 29.24 (SD±10.06) years. More than three-quarters (77.86%), of the participants were Orthodox Christians and 209 (51.99%) were married. The majority (74.44%) were Amhara by ethnicity and 78.36% of the participants were urban residents (Table 1).

### 3.2. Clinical and Obstetrical Characteristics of Participants

In this study, more than half (55.22%) of the participants were enrolled from health centers. Majority (83.58%) and three-quarters (74.63%) of the respondents had planned pregnancy and were attending ANC for the last pregnancy, respectively. Among those attending ANC, 177(44.03%) had three visits throughout their current pregnancy. Three hundred and six (76.12%) and 347 (86.32%) had spontaneous vaginal delivery and attended postnatal care, respectively (Table 2).

### 3.3. Knowledge of Participants about Infant Feeding

In this study, the overall participants who had good knowledge were 277 (68.91%). More than two-thirds (67.16%), just half (50.99%), and 44.8% of participants knew about mother to child transmission of HIV during breastfeeding, labor, and pregnancy, respectively (Table 3).

### 3.4. Attitude of Participants towards Infant Feeding

Participants who had favorable attitude towards recommended infant feeding were found to be 75.87%. The majority (85.57%) of participants agreed that exclusive breastfeeding for 6 months is nutritionally complete feeding for the infant (Table 4).

### 3.5. Feeding Practice of Participants

In this study majority (99.25%) of participants were practicing breastfeeding. Of those breastfeeding mothers, 61.9% and 66.67% breastfed their newborn within one hour after delivery and had experienced breastfeeding problem, respectively. This study found that 23.7% of mothers practiced infant feeding according to WHO recommendation. Two-thirds (67.92%) of participants feed their child less than eight times per day (Table 5).

## 4. Discussion

This study revealed the knowledge, attitude, and practice of HIV positive mothers on infant feeding options to children born from HIV infected mothers.

### 4.1. Knowledge on Infant Feeding

In this study, the overall level of good knowledge of participants was 68.91% (95% CI, 62.1–74.6%). This study found knowledge levels of participants greater than studies done in Jimma, Ethiopia, which is 38.8% [9], and Gaborone, Botswana [10]. This is encouraging result because, as Health Belief Model [11] described, the higher the perception of a mother about safe infant feeding practices, the more likely to practice it. This in turn reduces the risk of mother to child transmission (MTCT). But the finding of this study is lower than the finding in Hosanna, Ethiopia (92.8%) [12], and Tigray, Ethiopia (88.1%) [13].

In this study, 67.16% of the mothers answered “yes” to the question “Does HIV positive mothers transmit the virus to the baby during breastfeeding?” This is in line with 70% result from a similar study conducted in Gert Sibande district, South Africa [14].

This study also revealed that mothers who mentioned MTCT to be through pregnancy, labor, and breastfeeding were 54.2%. This finding is less than a study done in Gondar referral hospital (92.3%) [15]. This discrepancy could be from the fact that the current study included mothers from health centers and hospital while the former only included mothers who had follow-up in hospital where care and education are provided by better-qualified professionals.

Although there was a very high proportion of mothers who were aware of the possibility of an infected mother transmitting the infection to her child, only 129 (32%) said exclusive breastfeeding can reduce risk of diarrhea. This indicates the potential for introduction of feeding other than breast milk among mothers who do not agree on this statement.

### 4.2. Attitude on Infant Feeding

Regarding attitude on infant feeding, 75.87% of the mothers had favorable attitude towards safe infant feeding (95% CI, 69.8–81.7%). This is higher than a study done in southern Ethiopia which is 56.7% [16]. The time and geographical difference between these two studies, which might have effect on the attitude of participants, may justify the difference. On examining the responses of the study participants, nearly all (91.04%) agreed that breast milk is the ideal food for the baby less than six months of age. This is higher as compared to the findings in South Africa and Lesotho where 78.8% and 52% agreed that breast milk is the ideal food for the baby less than six months of age, respectively [17, 18]. This in part could be attributed to the fact that there is a decade time gap between the current study and the one conducted in South Africa. While the study conducted in Lesotho collected data from mothers having children up to six months, the current study collected data from mothers having children up to two years. This may have effect on maternal attitude on the long run with repeated exposure with health professionals.

### 4.3. Safe Infant Feeding Practice

The overall safe feeding practice consistent with WHO recommendation is 23.7% (95% CI, 17.1–29.7%). This is in line with a study on Ugandan women among whom 29% discussed their feeding to be consistent with WHO recommendations [19].

In this study the overall safe feeding practice based on WHO recommendation was much higher among age groups >35 years of age (76.92%) as compared to age group < 25 years of age (10.64%). This is supported by a study in Nigeria that showed older age was associated with increased rates of safe infant feeding practice [20]. The reason might be that those mothers with age > 35 years may have more children and more experience with infant feeding but young mothers may not be well experienced with regard to infant care including infant feeding options.

The proportion of mothers who practiced safe infant feeding was 27% and 16% among mothers who had good knowledge and poor knowledge about infant feeding, respectively. This is supported by the evidence from health belief model that says the higher the perception of people about health needs, the more likely they make decisions that promote health [11]. In other words, the higher the mother has knowledge about safe infant feeding practices, the more likely she practices them.

Among urban residents, the overall safe infant feeding practice was 26% but it was 12% among rural residents. This could be due to the fact that urban mothers are more informed about infant feeding options through the media and have good knowledge which increases the likelihood of practicing safe infant feeding. When it comes to attitude, 27.87% of mothers who had favorable attitude towards infant feeding practiced safe infant feeding while only 10.31% of mothers with unfavorable attitude practiced safe infant feeding. This finding is supported by a study in Addis Ababa that showed positive parental attitude to be a strong predictor of safe infant feeding practice [21]. This discrepancy can be attributed to the inclination that mothers have an infant feeding practice based on their attitude. In other words, mothers with favorable attitude are more likely to practice breastfeeding according to recommendations by health professionals while mothers with unfavorable attitude towards breastfeeding are less likely to follow recommendations.

Regarding exclusive breastfeeding, 23.06% of the mothers practiced exclusive breastfeeding for the first six months which is in line with a study in Addis Ababa, Ethiopia (30.6 %) [20]. But this result is lower than a result of a similar study done in southern Ethiopia where 48.2% of the mothers were practicing exclusive breastfeeding [16]. A study in India also showed that 57% of women practiced exclusive breastfeeding in the first six months of life [22]. This could be justified by the differences in the sociocultural nature of the study areas.

## 5. Conclusion

Even though the knowledge and attitude of participants about infant feeding are higher, the actual practice is low. Interventions should focus on bridging the gap between having good knowledge and favorable attitude and practicing of the recommendations.

## Figures and Tables

**Table 1:** Sociodemographic characteristics of HIV positive mothers attending ART in Gondar town health institutions, northwest Ethiopia, 2017(n=402).

**Variables **	**Frequency **	**Percent**
Age		
< 25	47	11.69
25-35	290	72.14
> 35	65	16.17
Religion		
Orthodox	313	77.86
Protestant	48	11.94
Muslim	41	10.20
Marital status		
Single	78	19.40
Married	209	51.99
Widowed	69	17.16
Divorce	46	11.45
Ethnicity		
Amhara	301	74.88
Oromo	35	8.71
Tigre	66	16.41
Educational status		
Unable to read and write	82	20.40
Able to Read and write	92	22.89
Grade 1-8	153	38.06
Grade 9 -12	50	12.44
College and above	25	6.21
Residence		
Urban	315	78.36
Rural	87	21.64
Occupation		
Government	163	40.55
employee	36	8.96
Housewife	95	23.63
Merchant	48	11.94
Student	21	5.22
Daily laborer	39	9.70
Nonemployed		
Monthly family income (Ethiopian Birr)		
< 1000	97	24.13
1000-2000	194	48.26
2001-3000	52	12.93
>3000	59	14.68
No of children		
< 3	241	59.95
≥ 3	161	40.05

**Table 2:** Clinical and obstetrical characteristics of HIV positive mothers attending ART in Gondar town health institutions, northwest Ethiopia, 2017(n=402).

**Variables **	**Frequency **	**Percent**
Setting in attending ART		
Hospital	180	44.78
Health centers	222	55.22
Age at first pregnancy		
< 20	41	10.20
20-25	125	31.09
26-30	199	49.50
>30	37	9.21
Number of live births		
1	63	15.67
2	144	35.82
3	130	32.34
>3	65	16.17
Was your last pregnancy planned?		
Yes	336	83.58
No	66	16.42
Did you attend ANC during the last pregnancy		
Yes	300	74.63
No	102	25.37
Number of ANC visit/s (n=300)		
One visit	24	5.97
Two visits	84	20.90
Three visits	177	44.03
Four or more visits	117	29.10
Place of last child delivery		
Hospital	140	34.83
Health center	207	51.49
Private clinic	15	3.73
Home	40	9.95
Mode of delivery		
Vaginal delivery	306	76.12
Caesarean section	96	23.88
Did you attend PNC during the last birth		
Yes	347	86.32
No	55	13.68

**Table 3:** Knowledge of HIV positive mothers attending ART in Gondar town health institutions, northwest Ethiopia, 2017(n=402).

**Variables **	**Frequency **	** Percent**
Overall knowledge level		
Good Knowledge	277	68.91
Poor knowledge	125	31.09
HIV transmitted through pregnancy, labor and breastfeeding		
Yes	218	54.2
No	184	45.8
Does HIV mother transmit the virus to her baby during labor		
Yes	205	50.99
No	155	38.56
Don't know	42	10.45
Does HIV mother transmit the virus to her baby during pregnancy		
Yes	177	44.03
No	180	44.78
Don't know	45	11.19
Does breast milk prevent childhood illness		
Yes	281	69.90
No	50	12.44
Don't know	71	17.66
Feeding only breast milk in the first six months helps boost the child immunity		
Yes	292	72.64
No	76	18.91
Don't know	34	8.45
Is it important to initiate breastfeeding within one hour after birth?		
Yes	267	66.42
No	60	14.93
Don't know	75	18.65
Can exclusive breastfeeding reduce the risk of diarrhea?		
Yes	129	32.09
No	144	35.82
Don't know	129	32.09
Growth patterns of exclusively breastfed infant/s differ from nonexclusively breastfed?		
Yes	288	71.64
No	47	11.69
Don't know	67	16.67
How long should exclusive breastfeeding be continued?		
< 6 months	60	14.93
6 months	301	74.87
> 6 months	41	10.20

**Table 4:** Attitude of HIV positive mothers attending ART in Gondar town health institutions, northwest Ethiopia, 2017(n=402).

**Variables **	**Frequency**	**Percent**
Overall level of attitude		
Favorable attitude	305	75.87
Unfavorable attitude	97	24.13
EBF (exclusive breastfeeding) for 6 months is the best choice for infant		
Agree	366	91.04
Disagree	36	8.96
EBF is not good since it transmits HIV		
Agree	297	73.88
Disagree	105	26.12
EBF for 6 months is nutritionally complete		
Agree	344	85.57
Disagree	58	14.43
Breastfeeding should be continued up to 2 years?		
Agree	196	48.76
Disagree	206	51.24
I do not accept EBF for fear of stigma due to HIV		
Agree	255	63.43
Disagree	147	36.57
Should breastfeeding be stopped when a child has diarrheal episode		
Agree	88	21.89
Disagree	314	78.11
Formula feeding better than breastfeeding?		
Agree	91	22.64
Disagree	311	77.36
Mixed feeding has a risk of HIV infection to infant/last child		
Agree	177	44.03
Disagree	225	55.97
Do you believe that breastfeeding causes changes in body shape for the mother?		
Agree	232	57.71
Disagree	170	42.29
Does breastfeeding increases mother-child bonding?		
Agree	369	91.79
Disagree	33	8.21
Bottle feeding is a good infant feeding option		
Agree	95	23.63
Disagree	307	76.37
Complimentary food after 6 months is the best choice		
Agree	333	82.84
Disagree	69	17.16

**Table 5:** Feeding practice of HIV positive mothers attending ART in Gondar town health institutions, northwest Ethiopia, 2017(n=402).

**Variables **	**Frequency **	**Percent**
Overall feeding practice		
Good practice	95	23.7%
Poor practice	307	76.3%
Do you breastfeed your child		
Yes	399	99.25
No	3	0.75
Initiation of breastfeeding (n=399)		
Immediately after delivery	67	16.79
Within one hour after delivery	247	61.90
After one hour of delivery	85	21.31
Have you experienced any breastfeeding problem (n=399)		
Yes	266	66.67
No	133	33.33
What was the breastfeeding problem (n=266)		
Not enough milk	240	90.23
Nipple crack	16	6.01
Baby mouth ulcer	10	3.76
What did you feed your baby within the first three days (n=399)		
Only breastfeed	300	75.19
Water and sugar	67	16.79
Butter	32	8.02
Duration of breastfeeding (n=399)		
Birth to 6 months	177	44.36
12 months	106	26.57
18-24 months	116	29.07
Frequency of breastfeeding/day (n=399)		
< 8 times	271	67.92
≥ 8 times	128	32.08
Duration of Exclusive Breastfeeding (n=399)		
1-2 months	100	25.06
3-5 months	207	51.88
6 months	92	23.06

## Data Availability

The data used to support the findings of this study are included within the article.

## Acronyms

AFASS: Acceptable, Feasible, Affordable, Sustainable, and Safe
AIDS: Acquired Immune Deficiency Syndrome
ANC: Antenatal Care
ART: Antiretroviral therapy
EBF: Exclusive breastfeeding
EDHS: Ethiopian Demographic and Health Survey
HIV: Human Immunodeficiency Virus
KAP: Knowledge, attitude, and practice
MBF: Mixed breastfeeding
MTCT: Mother to child transmission
NGO: Nongovernmental Organization
PMTCT: Prevention of mother to child transmission
SPSS: Statistical Package for Social Sciences
UNAIDS: United Nation Program on HIV/AIDS
UNFPA: United Nation Fund population
WHO: World Health Organization.
